# Supplementary material for: Cross-linguistically consistent semantic and syntactic annotation of child-directed speech
Source: Lang Resour Eval. 2024 May 15;59(2):727–76. doi: 10.1007/s10579-024-09734-y (PMC12086124; doi:10.1007/s10579-024-09734-y)
Supplement: Supplementary file 1 — (PDF 642 KB) [file 10579_2024_9734_MOESM1_ESM.pdf]

1. Supplementary material

1.1 Longitudinal analysis

For most dependencies we observe no regular change in frequency as the child gets older. The constructions for which a trend was detected are discussed in the main paper. Here we present the plots of frequency over time for all dependencies in both corpora. Each graph combines data from the Adam and Hagar corpora to allow for an easier comparison across languages and ages. Notice that the Hagar corpus starts at an earlier age and that the sessions were much more frequent than in the Adam corpus, leading to more jagged plots.

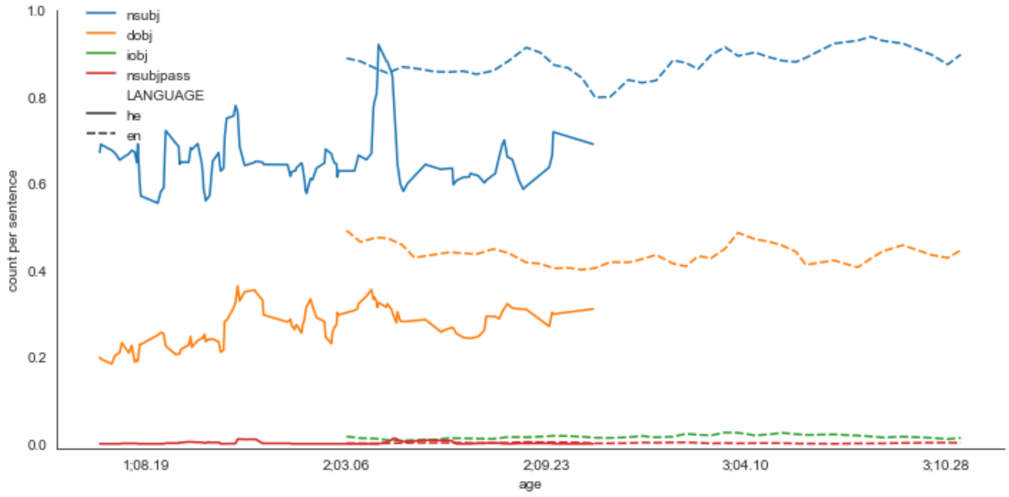

(a) Core arguments

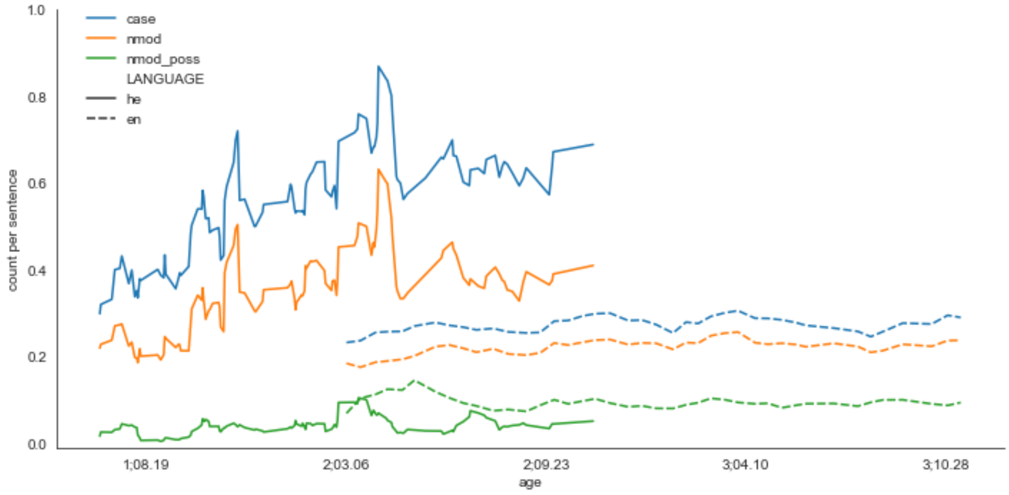

(b) Nominal modifiers, part 1

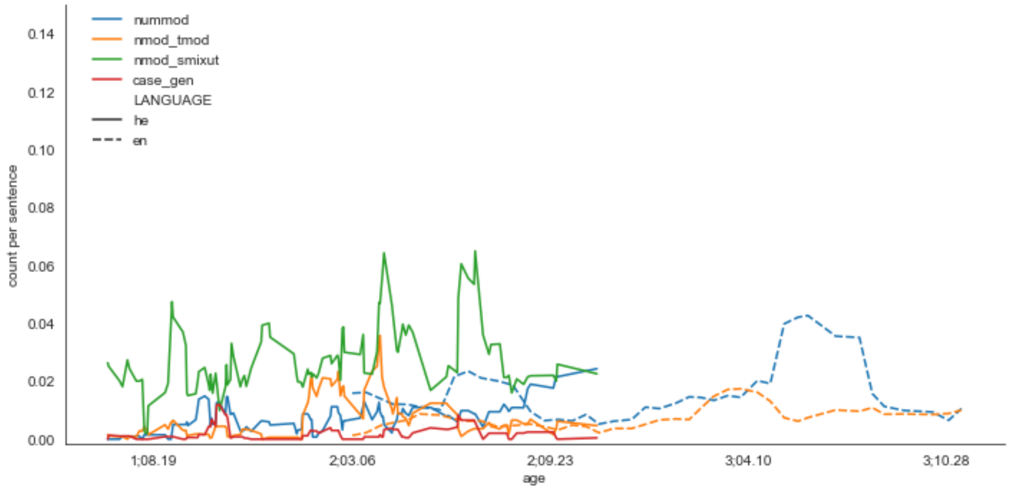

(c) Nominal modifiers, part 2

Figure 1: Proportion of sentences containing given dependency per session in the Adam (dashed) and Hagar (solid) corpora; frequencies are smoothed over 5 sessions.

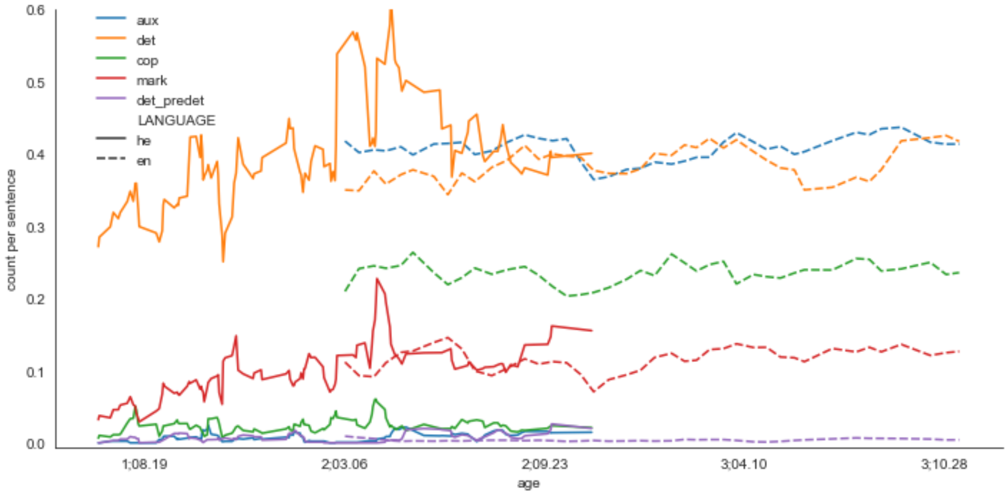

(a) Determiners and purely syntactic relations

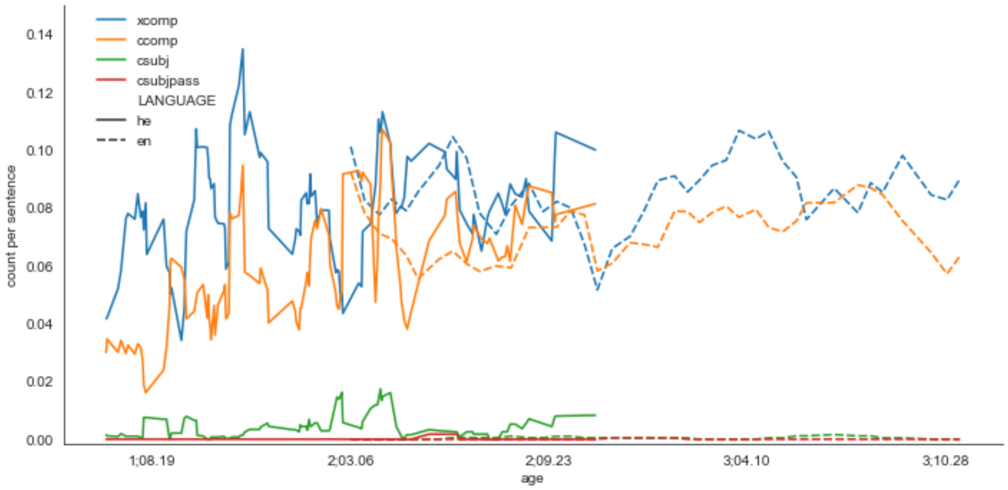

(b) Clausal arguments

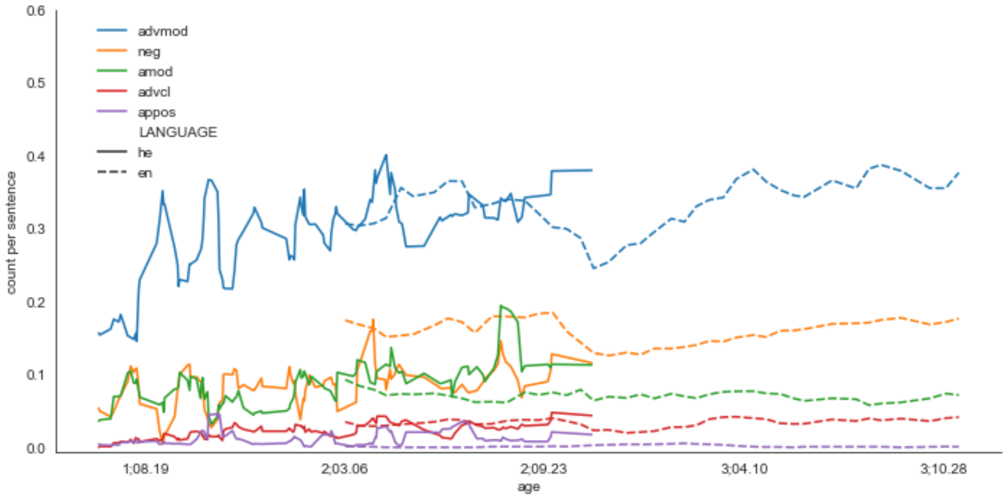

(c) Modifiers of nominals

Figure 2: Proportion of sentences containing given dependency per session in the Adam (dashed) and Hagar (solid) corpora; frequencies are smoothed over 5 sessions.

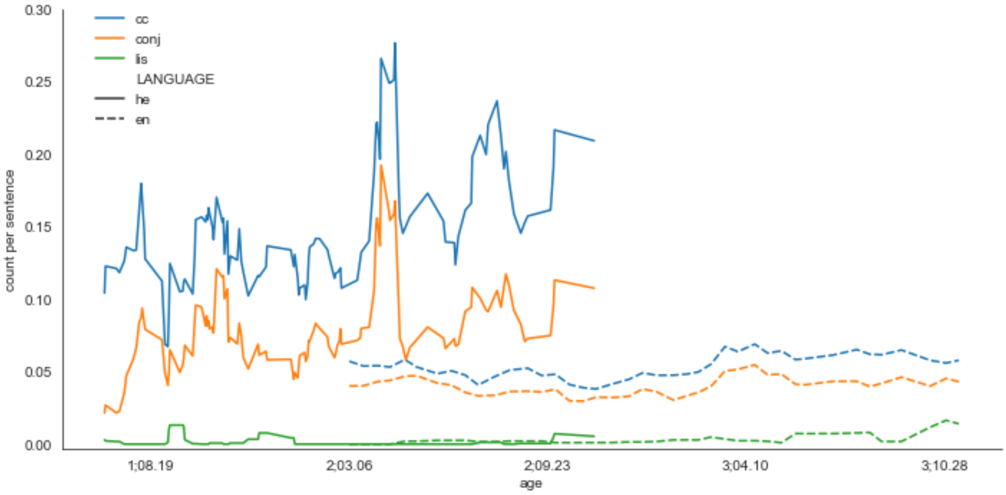

(a) Conjunction

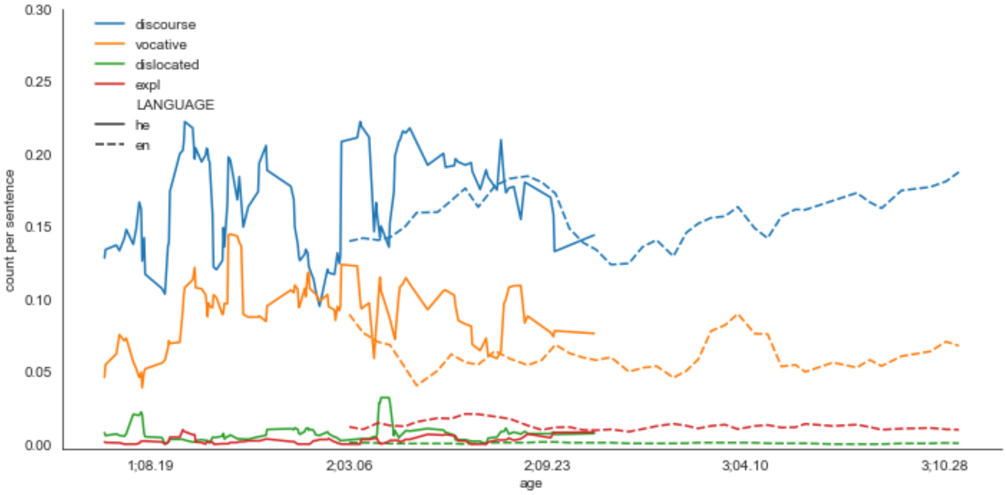

(b) Discursive relations, part 1

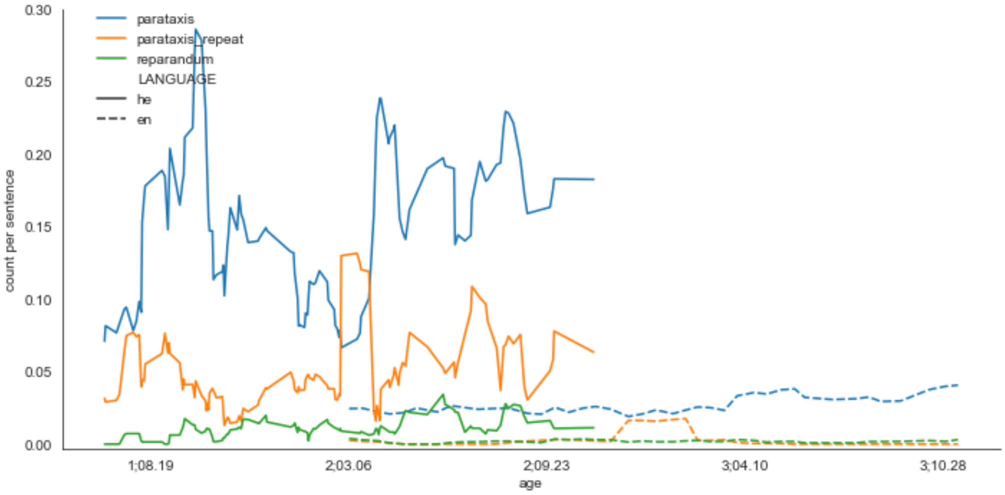

(c) Discursive relations, part 2

Figure 3: Proportion of sentences containing given dependency per session in the Adam (dashed) and Hagar (solid) corpora; frequencies are smoothed over 5 sessions.

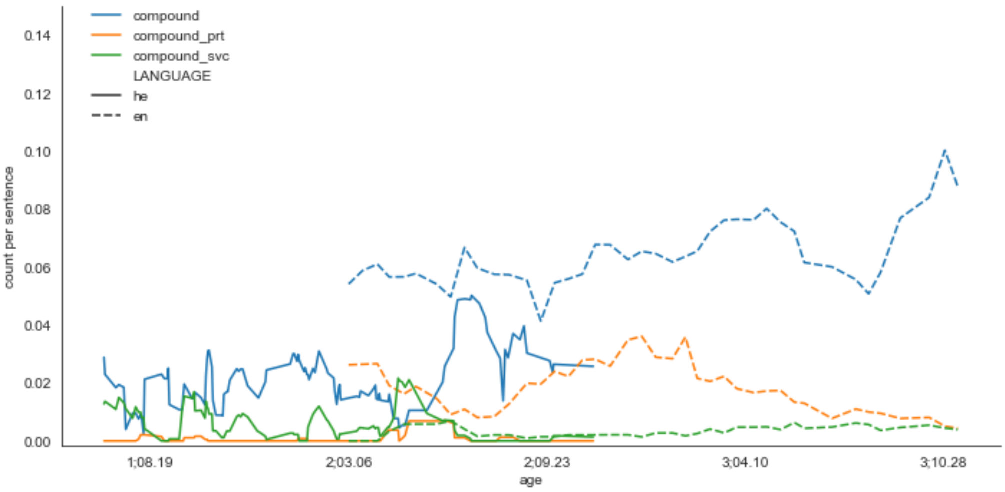

(a) Compounding, part 1

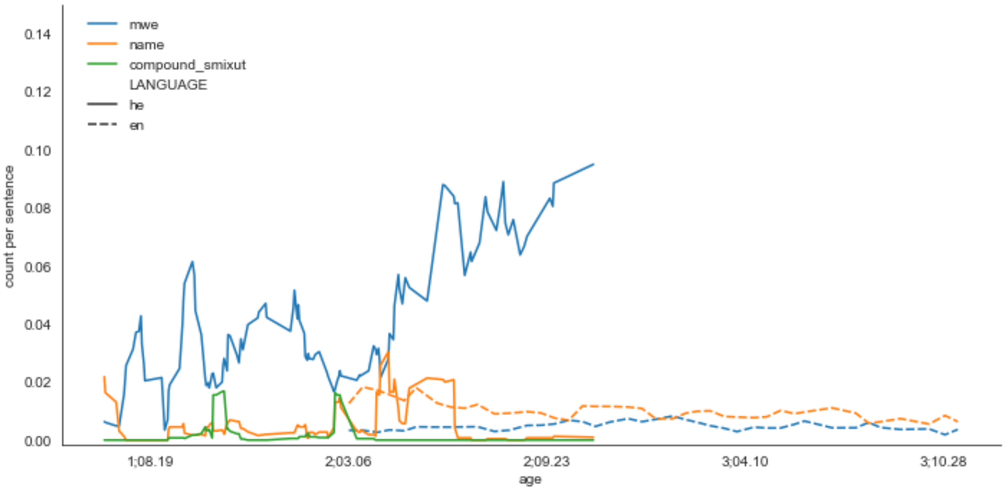

(b) Compounding, part 2

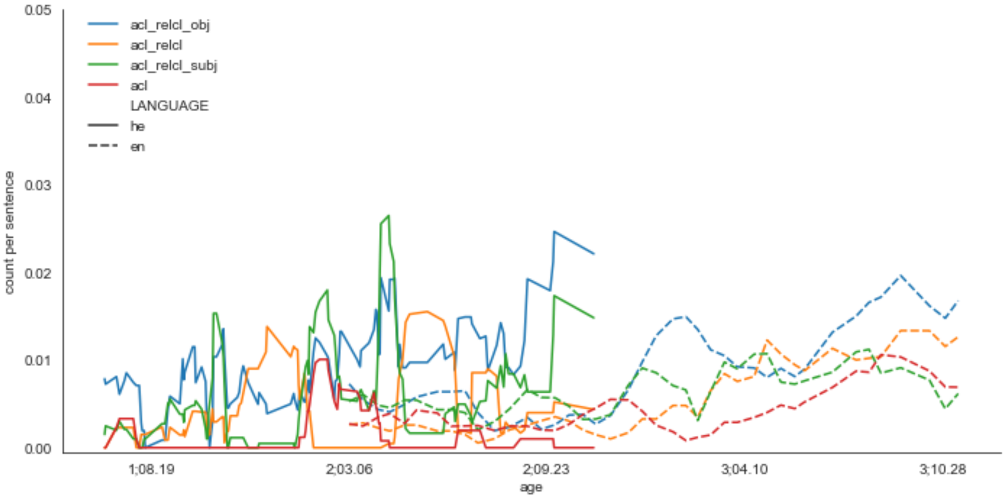

(c) Relative clause

Figure 4: Proportion of sentences containing given dependency per session in the Adam (dashed) and Hagar (solid) corpora; frequencies are smoothed over 5 sessions.

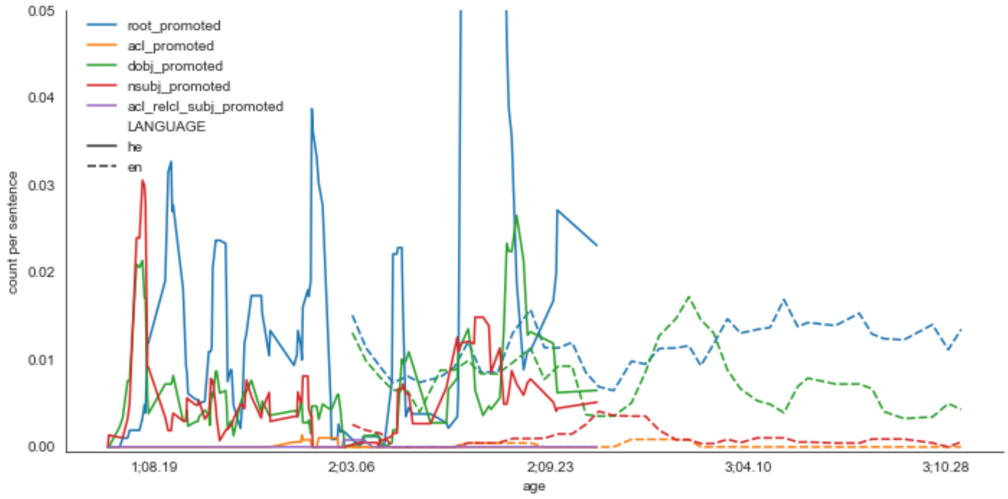

(a) Ellipsis, part 1

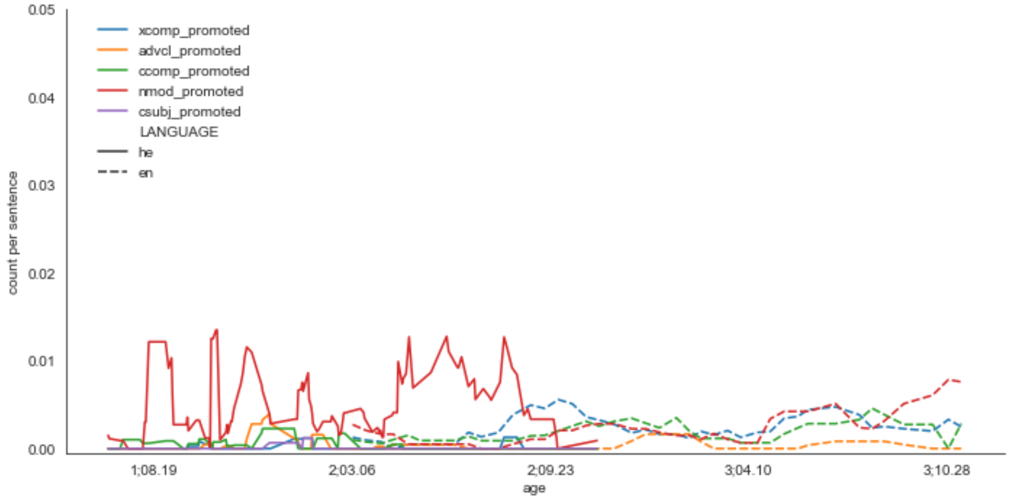

(b) Ellipsis, part 2

Figure 5: Proportion of sentences containing given dependency per session in the Adam (dashed) and Hagar (solid) corpora; frequencies are smoothed over 5 sessions.
